# DNA barcoding and evaluation of genetic diversity in Cyprinidae fish in the midstream of the Yangtze River

**DOI:** 10.1002/ece3.2060

**Published:** 2016-03-17

**Authors:** Yanjun Shen, Lihong Guan, Dengqiang Wang, Xiaoni Gan

**Affiliations:** ^1^ The Key Laboratory of Aquatic Biodiversity and Conservation of Chinese Academy of SciencesInstitute of HydrobiologyChinese Academy of SciencesWuhan430072HubeiChina; ^2^ University of Chinese Academy of SciencesBeijing100039China; ^3^ College of Life Science and TechnologyXinxiang Medical UniversityHe'nan Xinxiang453003China; ^4^ The Key Laboratory of Freshwater Biodiversity ConservationMinistry of AgricultureYangtze River Fisheries Research InstituteChinese Academy of Fishery SciencesWuhanChina

**Keywords:** Cyprinidae, cytochrome c oxidase subunit I, DNA barcoding, identification, Kimura's two‐parameter

## Abstract

The Yangtze River is the longest river in China and is divided into upstream and mid‐downstream regions by the Three Gorges (the natural barriers of the Yangtze River), resulting in a complex distribution of fish. Dramatic changes to habitat environments may ultimately threaten fish survival; thus, it is necessary to evaluate the genetic diversity and propose protective measures. Species identification is the most significant task in many fields of biological research and in conservation efforts. DNA barcoding, which constitutes the analysis of a short fragment of the mitochondrial cytochrome c oxidase subunit I (COI) sequence, has been widely used for species identification. In this study, we collected 561 COI barcode sequences from 35 fish from the midstream of the Yangtze River. The intraspecific distances of all species were below 2% (with the exception of *Acheilognathus macropterus* and *Hemibarbus maculatus*). Nevertheless, all species could be unambiguously identified from the trees, barcoding gaps and taxonomic resolution ratio values. Furthermore, the COI barcode diversity was found to be low (≤0.5%), with the exception of *H. maculatus* (0.87%), *A. macropterus* (2.02%) and *Saurogobio dabryi* (0.82%). No or few shared haplotypes were detected between the upstream and downstream populations for ten species with overall nucleotide diversities greater than 0.00%, which indicated the likelihood of significant population genetic structuring. Our analyses indicated that DNA barcoding is an effective tool for the identification of cyprinidae fish in the midstream of the Yangtze River. It is vital that some protective measures be taken immediately because of the low COI barcode diversity.

## Introduction

The Yangtze River is the longest river in China. It originates from the Tibetan Plateau at an elevation higher than 5,000 m, flows first south, then north and northeast, and finally east to reach the coast, 6,300 km away (Chen et al. 2001). The Yangtze River is divided into upstream and mid‐downstream regions by the Three Gorges (TG; the natural barriers of the Yangtze River), resulting in a complex distribution of fish (Wang et al. 2004). Nearly 300 species are estimated to live in the tributaries and main stream. Cyprinidae are the predominant family of fish in the Yangtze River, representing 54.02% of all species (Institute of Hydrobiology 1976). However, many cyprinidae fish populations have been disturbed by human activities. For example, the number of *Coreius guichenoti* in the Yangtze River has declined significantly due to environmental pollution, overexploitation, and construction of electrical projects (Duan et al. 2002). Today the situation may be even worse, especially after the construction of the Three Gorges Dam (TGD) in the middle of the Yangtze River in 2009. The environments for habitats and spawning fields have dramatically changed, particularly for downstream fish; these changes may ultimately threaten the survival of fish because the TGD blocks natural fish migration patterns. Therefore, it is necessary to evaluate the genetic diversity and propose protective measures for cyprinidae fish in the midstream of the Yangtze River.

Species identification is the most significant task in many fields of biological research and conservation efforts. Traditional morphological identification is not fully effective for eggs, fry, and adults lacking distinctive morphological characteristics. Moreover, the number of specialists in alpha taxonomy is insufficient for convenient and complex morphological identification (Carvalho et al. 2011, 2015). Thus, rapid, reliable, and reproducible molecular tests to identify fish species are needed in many areas (Rasmussen and Morrissey 2009; Steinke et al. 2009). One proposed method is DNA barcoding, which uses the mtDNA gene cytochrome oxidase subunit I (COI) as a global DNA barcoding identification system for animals (Hebert et al. 2003a,b). Sequences for the same species are generally considered to be correctly identified when they form a monophyletic cluster on a neighbor‐joining (NJ) tree with intraspecific distances that are below a given threshold (Srivathsan and Meier 2012). At present, this approach has proven to be highly efficient and reliable in many fish groups (Ward et al. 2005; Hubert et al. 2008, 2010; Rock et al. 2008; Keskin et al. 2013; Loh et al. 2014) and is regularly used for a variety of applications, such as fishery management, biodiversity assessments and conservation (Triantafyllidis et al. 2011; Weigt et al. 2012; Keskin et al. 2013; Loh et al. 2014; Shen et al. 2016).

Species identification with DNA barcodes is reliable only if a significant difference between the average intraspecific and the average interspecific genetic distances can be consistently detected (Hebert et al. 2003a,b, 2004; Ward et al. 2005). The use of Kimura's two‐parameter (K2P) model (Kimura 1980) in DNA barcoding studies began with (Hebert et al. 2003a,b) and is now widely used to assign an unknown specimen to a known species, to detect novel sequences, and to determine whether an unknown specimen is a distinct new species (Pereira et al. 2011; Hsu et al. 2013). K2P is computationally fast and yields consistent results for many species that exhibit the necessary disparity between intra‐ and interspecific variation. However, the use of the K2P distance in barcode analyses has been challenged and the p‐distance has been proposed to be a better model (Collins et al. 2012; Srivathsan and Meier 2012). The lack of overlap between intra‐ and interspecific variation (dubbed the “barcoding gap”) has been deemed to be of paramount importance for the accuracy and reliability of barcode genes (Meyer and Paulay 2005) and can be influenced by distance models (Collins et al. 2012; Srivathsan and Meier 2012). Hebert et al. (2004) have defined the “barcoding gap” as the existence of at least a 10 times greater average interspecific distance than average intraspecific genetic distance. Therefore, in the present study, both the K2P and p‐distance models were used in the barcoding gap analysis.

The present study explored the utility of the DNA barcoding approach as a molecular technique for the identification of Cyprinidae fish in the midstream of the Yangtze River and evaluated the identification success rates based on the K2P and p‐distance models. Furthermore, a preliminary genetic diversity analysis of COI was performed for some fish species to tentatively provide important information for the conservation of the Cyprinidae fish resource in the midstream of the Yangtze River.

## Materials and Methods

The experiments were performed in accordance with the Ethics Committee of the Institute of Hydrobiology at the Chinese Academy of Sciences. The policies were enacted according to the Chinese Association for Laboratory Animal Sciences and the Institutional Animal Care and Use Committee (IACUC) protocols.

### Sample collection and morphological identification

**Figure 1:**
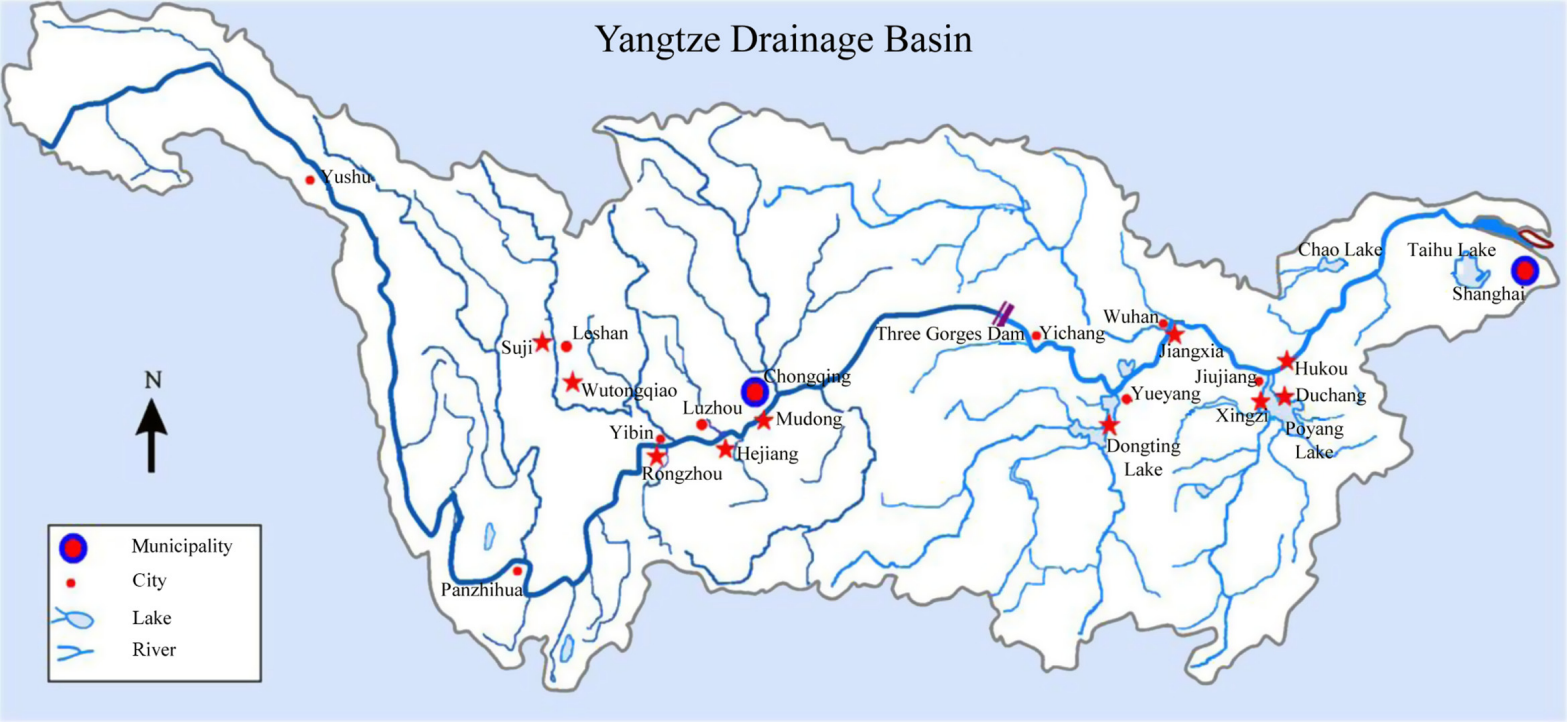
Sampling sites of cyprinidae fish represented by red five‐pointed stars from the midstream of the Yangtze River.

In 2011, a total of 561 samples from 35 species, 25 genera and eight subfamilies of cyprinidae (Table S1) were collected from ten different sites in the midstream of the Yangtze River (Fig. 1). In most cases, the specimens were obtained from research vessel trawling surveys conducted in multiple zones of the Yangtze River to inform fishery management of the status of fish stocks. Morphological identification was performed in situ by visual inspection, and the fish were taxonomically classified by employing standard guides referencing Fauna Sinica (Chen 1998) and the FishBase databases (Froese and Pauly 2015). As many individuals per species as possible were obtained for this study. However, in some cases, only one or two individuals per region per species could be collected, which precluded accurate calculations of population parameters (e.g., genetic diversity). Therefore, population estimates were not made for these species, but this shortcoming is unlikely to have affected the main conclusions of this study. Tissue samples were obtained and immediately preserved in 100% ethanol for DNA extraction. All whole fish samples were stored as voucher samples in a 10% formaldehyde solution and deposited in the Museum of the Institute of Hydrobiology at the Chinese Academy of Sciences. All sample sequences were identified through a BLAST search of the NCBI (National Center for Biotechnology Information) and BOLD (Barcode of Life) databases (Ratnasingham and Hebert 2007).

### DNA extraction, amplification and sequencing

Total DNA was extracted from a fin clip or muscle sample by standard salt extraction (Reisfeld et al. 1971) and verified using 1.0% agarose gel electrophoresis. A partial fragment from the 5′ end of the mitochondrial COI gene was amplified using various combinations of the following fish‐specific primers: FishF1‐TCAACCAACCACAAAGACATTGGAC, FishF2‐TCGACTAATCATAAAGATATCGGCAC, FishR1‐TAGACTTCTGGGTGGCCAAAGAATCA, and FishR2‐ACTTCAGGGTGACCGA AGAATCAGAA (Ward et al. 2005).

The 30 *μ*L polymerase chain reaction (PCR) mixtures contained 21.125 *μ*L of sterilized ultrapure water, 3.0 *μ*L of 10× PCR buffer (including MgCl_2_), 1.5 *μ*L of each primer (10 mmol/L), 1.5 *μ*L of dNTPs (2.5 mmol/L each), 0.375 *μ*L of Taq DNA polymerase (2.5 U/*μ*L, TaKaRa Bio, Shanghai, China), and 1.0 *μ*L of the DNA template (50–100 ng/*μ*L). The PCR amplification conditions were as follows: 94°C for 5 min, 32 cycles at 94°C for 30 s, 53°C for 30 s and 72°C for 1 min, and a final extension at 72°C for 10 min. The PCR products were visualized on a 1.2% agarose gel. Successful amplifications were isolated from the gels, purified with a DNA Gel Extraction Kit (Tiangen, Wuhan, China), and sequenced on an ABI3730 XL DNA System.

### Data analysis

The sequence chromatograms and alignments were visually inspected and verified using the DNASTAR Lasergene package (DNASTAR, Inc., Madison, WI, USA). Sequences were aligned and trimmed to the same length using the software package MEGA 5.0 (Tamura et al. 2011), and all the aligned sequences were translated into amino acids to confirm the effectiveness of the sequences and to detect the presence of nuclear DNA pseudogenes, insertions, deletions or stop codons. The COI sequence similarities were obtained by aligning the sequences from the 35 species to homologous fragment sequences in the NCBI and BOLD databases to evaluate the accuracy of the morphological identification. Similarly to Wong (Wong and Hanner 2008), we used a general rule that defined a top match with a sequence similarity of at least 97% to indicate a potential species identification and 3% as a relatively loose criterion.

*Misgurnus anguillicaudatus* (GenBank: JN177217.1) and *Paramisgurnus dabryanus* (GenBank: JN177218.1) were chosen as the outgroups. All COI sequences were converted into haplotype data. Then, sequence comparisons, pairwise genetic distance calculations, and NJ tree analysis were implemented using MEGA 5.0 with the default parameters and 10,000 bootstrap replicates. The average values of the K2P distances and p‐distances obtained for the conspecific and congeneric divergences were applied in the calculation of the taxonomic resolution ratio (TRR), which was defined as the quotient between the congeneric and conspecific divergences (Costa et al. 2007). The DNA barcoding gap, which was the maximum intraspecific distance of each species against its minimum distance to the nearest neighbor, was calculated for all species.

We constructed a maximum‐likelihood (ML) tree using PhyML 3.0 (Guindon and Gascuel 2003) with 10,000 replicates, and the most appropriate TrN + I + G substitution model was identified using Modeltest 3.7 (Posada and Crandall 1998). A Bayesian tree was also established by using MrBayes 3.1.2 (Ronquist and Huelsenbeck 2003) with 5,000,000 replicates using the TrN + I + G model identified by jModelTest 0.1.1 (Posada 2008). In all trees, species branches with multiple haplotypes were merged into one branch. Bootstrap values below 50% are not shown, and the number of multisamples is indicated by the form “*n*=”.

Genetic diversity is reflected by the measurements of nucleotide diversity (*π*) and haplotype diversity (*h*) (Nei 1987). Therefore, we computed the number of haplotypes, nucleotide diversity (*π*) and haplotype diversity (*h*) for species with sample numbers greater than five and populations divided according to their locations upstream and downstream of the TG using DnaSP 5.0 (Librado and Rozas 2009).

## Results

Ten of the 35 species evaluated were endemic to the Yangtze River. The number of individuals per species ranged from one to 56 (mean 16), with six species represented by less than three individuals and four species (*Opsariichthys bidens*,* Procypris rabaudi*,* Rhodeus ocellatus*, and *Saurogobio dumerili*) represented by only one specimen. Three species (*Saurogobio gracilicaudatus*,* Ancherythroculter kurematsui*, and *Hemiculter tchangi*) were barcoded for the first time, and 11 species were represented by only one sampling site (Table S1).

### Amplification and sequencing

Low‐quality sequences (double peaks, short fragments, and background noise) that may have represented pseudogenes were not detected. Ultimately, the aligned sequences, which contained no insertions, deletions or stop codons, indicated that all amplified sequences were functional mitochondrial COI sequences. In total, 561 COI sequences were successfully amplified in this study and were submitted to the BOLD (under the project title “CJDB DNA barcoding of the Yangtze River: 35 kinds of cyprinidae”) and GenBank databases (Table S1). The COI genes of each species were aligned to yield a final sequence fragment of 624 bp that contained 241 variable sites, 233 of which were parsimony informative. Moreover, our morphological identification results matched the BLASTN annotations of the NCBI and BOLD databases, with at least 97% similarity except for three species (*S. gracilicaudatus*,* A. kurematsui*, and *H. tchangi*), which matched at only the genus level because no sequence information for these three species was available in the database (Table 1).

**Table 1:** Comparisons of COI sequences similarity in NCBI and BOLD database, scientific name from biological characters

Scientific name	NCBI			BOLD	
	Max score	ID (%)	Species name	Similarity (%)	Species name
*Abbottina obtusirostris*	1136	99	*Abbottina obtusirostris*	No	No match
*Abbottina rivularis*	1147	99	*Abbottina rivularis*	100	*Abbottina rivularis*
*Carassius auratus*	1153	100	*Carassius auratus*	100	*Carassius auratus*
*Coreius heterodon*	1153	100	*Coreius heterodon*	100	*Coreius heterodon*
*Ctenopharyngodon idellus*	1153	100	*Ctenopharyngodon idellus*	100	*Ctenopharyngodon idellus*
*Culter alburnus*	1153	100	*Culter alburnus*	100	*Culter alburnus*
*Culter mongolicus*	1153	100	*Culter mongolicus*	100	*Culter mongolicus*
*Cultrichthys erythropterus*	1142	99	*Cultrichthys erythropterus*	No	No match
*Cyprinus carpio*	1153	100	*Cyprinus carpio*	100	*Cyprinus carpio*
*Elopichthys bambusa*	1002	97	*Elopichthys bambusa*	No	No match
*Hemiculter bleekeri*	1142	99	*Hemiculter bleekeri*	No	No match
*Hemiculter leucisculus*	1153	100	*Hemiculter leucisculus*	100	*Hemiculter leucisculus*
*Hypophthalmichthys molitrix*	1147	99	*Hypophthalmichthys molitrix*	99.8	*Hypophthalmichthys molitrix*
*Hypophthalmichthys nobilis*	1153	100	*Hypophthalmichthys nobilis*	100	*Hypophthalmichthys nobilis*
*Opsariichthys bidens*	1098	98	*Opsariichthys bidens*	97.8	*Opsariichthys bidens*
*Procypris rabaudi*	1153	100	*Procypris rabaudi*	99.4	*Procypris rabaudi*
*Pseudobrama simoni*	1147	100	*Pseudobrama simoni*	100	*Pseudobrama simoni*
*Pseudolaubuca engraulis*	1153	100	*Pseudolaubuca engraulis*	100	*Pseudolaubuca engraulis*
*Pseudolaubuca sinensis*	1038	97	*Pseudolaubuca sinensis*	No	No match
*Rhinogobio typus*	1147	99	*Rhinogobio typus*	97.1	*Rhinogobio typus*
*Rhodeus ocellatus*	1142	99	*Rhodeus ocellatus*	99.7	*Rhodeus ocellatus*
*Sarcocheilichthys sinensis*	1092	98	*Sarcocheilichthys sinensis*	97.9	*Sarcocheilichthys sinensis*
*Saurogobio dabryi*	1142	99	*Saurogobio dabryi*	98.5	*Saurogobio dabryi*
*Saurogobio dumerili*	1147	99	*Saurogobio dumerili*	No	No match
***Saurogobio gracilicaudatus***	**821**	**90**	***Saurogobio***	**No**	**No match**
*Squalidus argentatus*	1147	99	*Squalidus argentatus*	99.8	*Squalidus argentatus*
*Ancherythroculter nigrocauda*	1037	97	*Ancherythroculter nigrocauda*	No	No match
*Squaliobarbus curriculus*	1153	100	*Squaliobarbus curriculus*	100	*Squaliobarbus curriculus*
*Parabramis pekinensis*	1153	100	*Parabramis pekinensis*	No	No match
*Xenocypris argentea*	1147	100	*Xenocypris argentea*	100	*Xenocypris argentea*
*Rhinogobio cylindricus*	1142	99	*Rhinogobio cylindricus*	No	No match
***Ancherythroculter kurematsui***	**998**	**96**	***Ancherythroculter***	**No**	**No match**
***Hemiculter tchangi***	**1064**	**96**	***Hemiculter***	**96.9**	***Hemiculter***
*Hemibarbus maculatus*	1147	99	*Hemibarbus maculatus*	99.8	*Hemibarbus maculatus*
*Acheilognathus macropterus*	1153	100	*Acheilognathus macropterus*	99.7	*Acheilognathus macropterus*

Species with bold font have low match values.

### Genetic distance and barcoding gap

**Figure 2:**
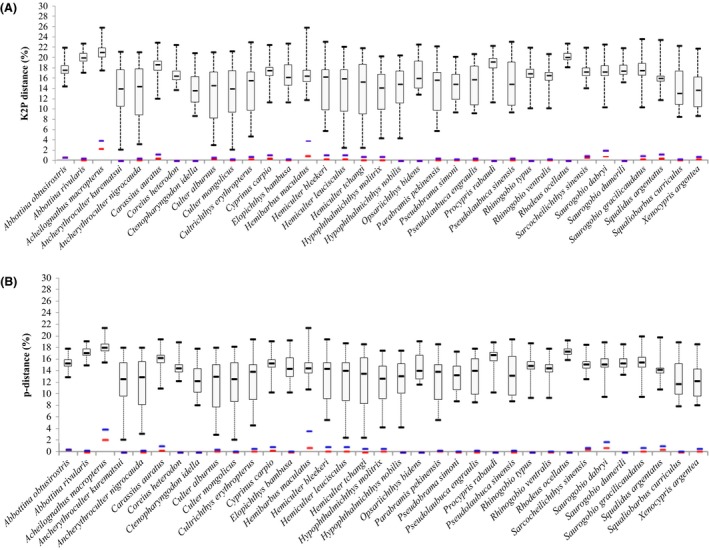
DNA barcoding gaps for all of the species based on the (A) K2P and (B) p‐distance models. Median interspecific distances with maximum and minimum values are represented by the upper and lower bars, respectively. Blue line: Maximum intraspecific distance; Red line: Mean intraspecific distance.

The K2P distances and p‐distances were compared at the intraspecific and intragenus levels. The intraspecific K2P distances were less than 3.81% and the mean distance was 0.36%, whereas the intraspecific p‐distances were less than 3.70% and the mean distance was 0.35%. The intragenus K2P distances ranged from 2.47% to 18.25% and the mean distance was 7.05%, whereas the intragenus p‐distances ranged from 2.42 to 15.78% and the mean distance was 6.60%. Genetic divergence increased with the increase in the taxonomic level; thus, the TRR values for the two models were 19.67 and 18.33, respectively (Table 2). The maximum K2P distances of all species were less than 2%, with the exception of *Acheilognathus macropterus* and *Hemibarbus maculatus*, which were both 3.81%. The maximum p‐distances of all species were also less than 2%, with the exception of *A. macropterus* and *H. maculatus*, which were 3.54 and 3.70%, respectively (Fig. 2). Nevertheless, both species were unambiguously identified using COI barcoding because the K2P distances to their nearest neighbors were 18.86% for *A. macropterus* and 13.51% for *H. maculatus*, whereas the p‐distances were 15.38 and 10.73%, respectively.

**Table 2:** Distance summary based on K2P and p‐distance models within species and genus levels

Model	Level	Min dist (%)	Mean dist (%)	Max dist (%)	SE dist (%)	TRR^a^
K2P	Within species	0.00	0.36	3.81	0.01	19.67
	Within genus	2.47	7.08	18.25	0.04	
p‐Distance	Within species	0.00	0.35	3.70	0.01	18.33
	Within genus	2.42	6.60	15.78	0.03	

a TRR, taxonomic resolution ratio (see Data analysis in Materials and methods).

The species discrimination power of DNA barcoding was demonstrated by the barcoding gaps that were drawn for all species on the basis of the K2P distances and p‐distances shown in Figure 2. Because the latter value was always higher than the former, overlaps were not detected in all species.

### Tree analyses

**Figure 3:**
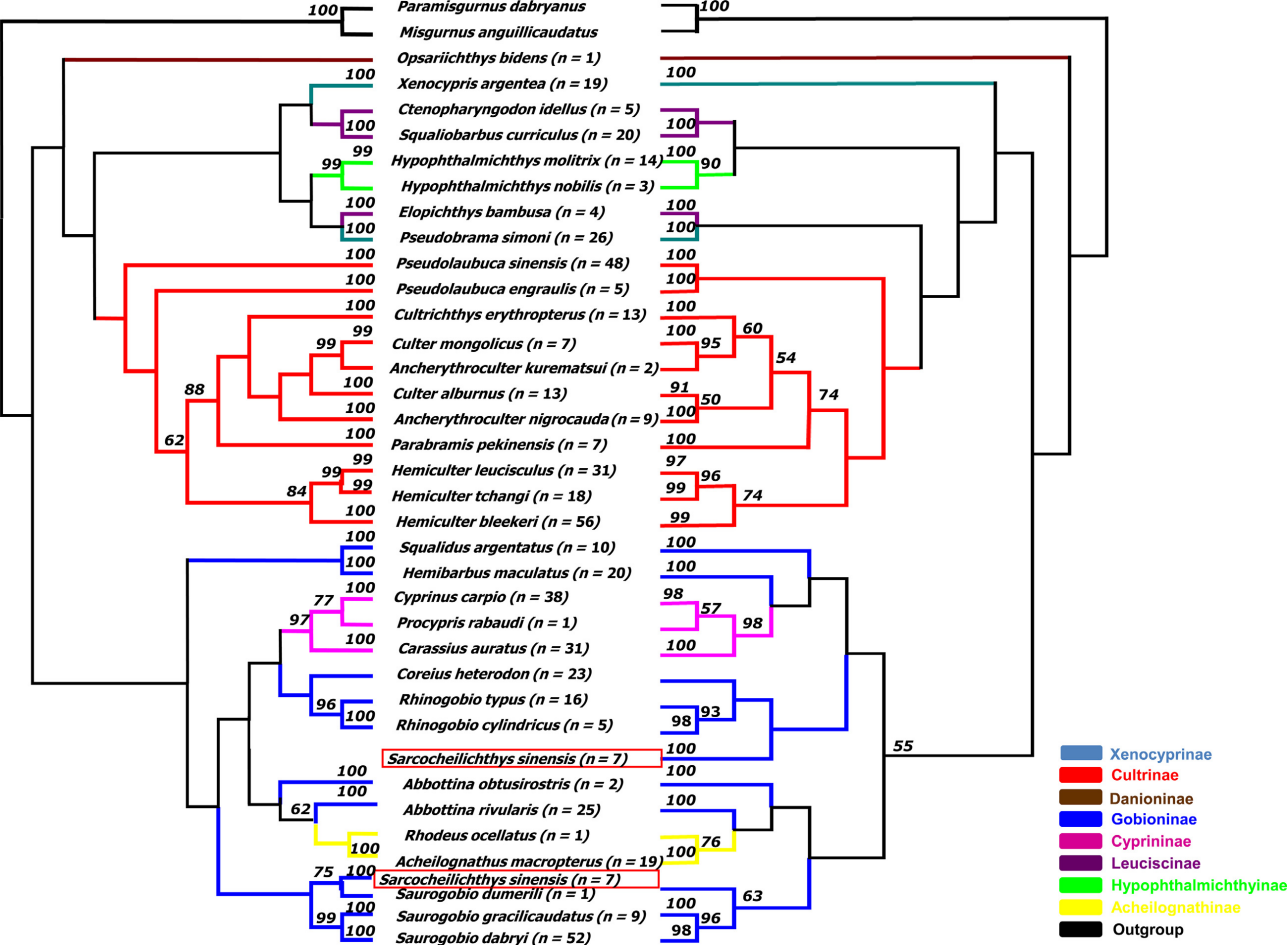
Neighbor‐joining tree constructed with MEGA based on the K2P model (left) and the maximum‐likelihood tree constructed with PhyML (right). Bootstrap values greater than 50 are shown. *n*: the number of samples. Each color represents a subfamily.

**Figure 4:**
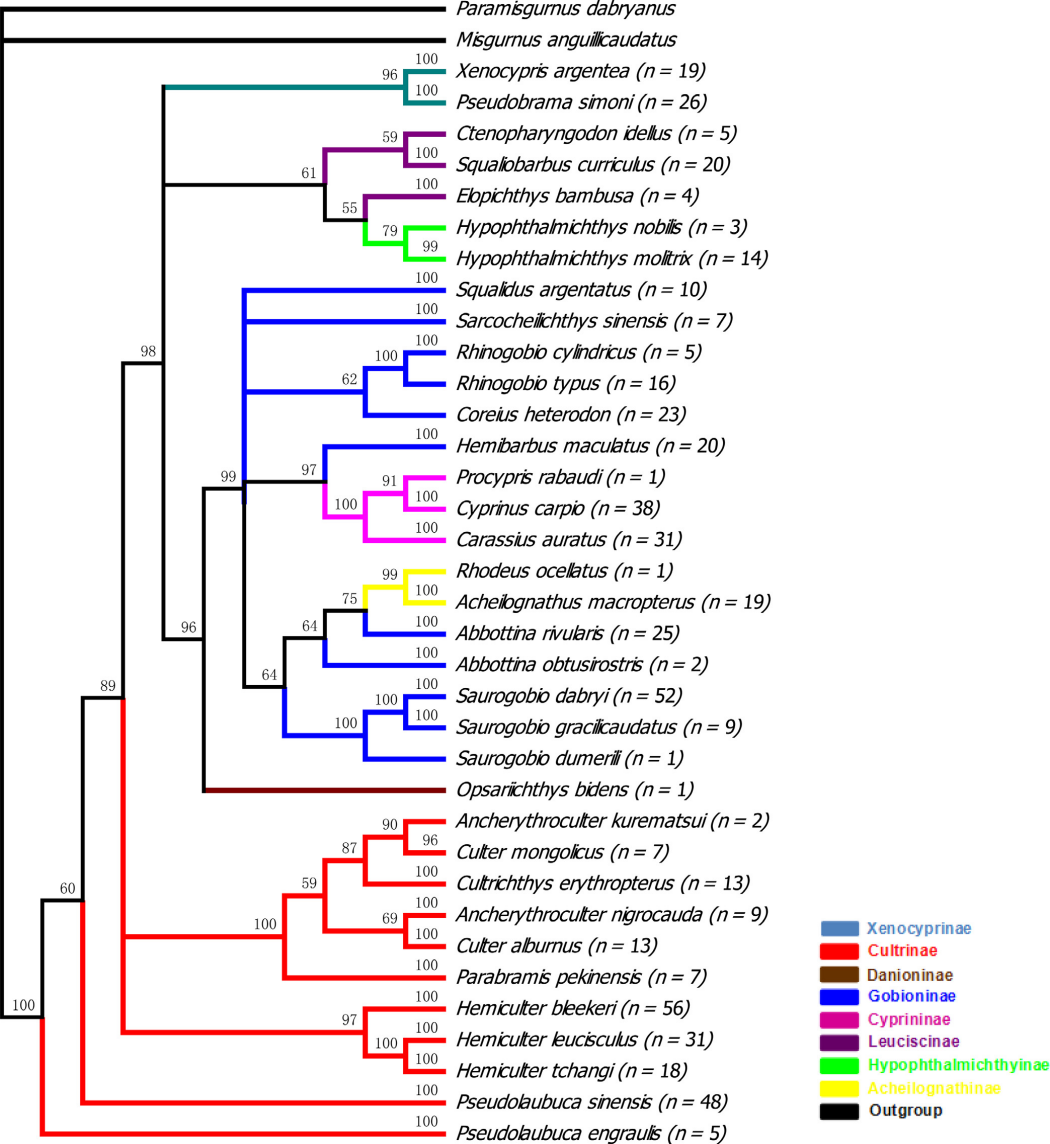
Bayesian tree constructed with MrBayes. Bootstrap values greater than 50 are shown. *n*: the number of samples. Each color represents a subfamily.

**Figure 5:**
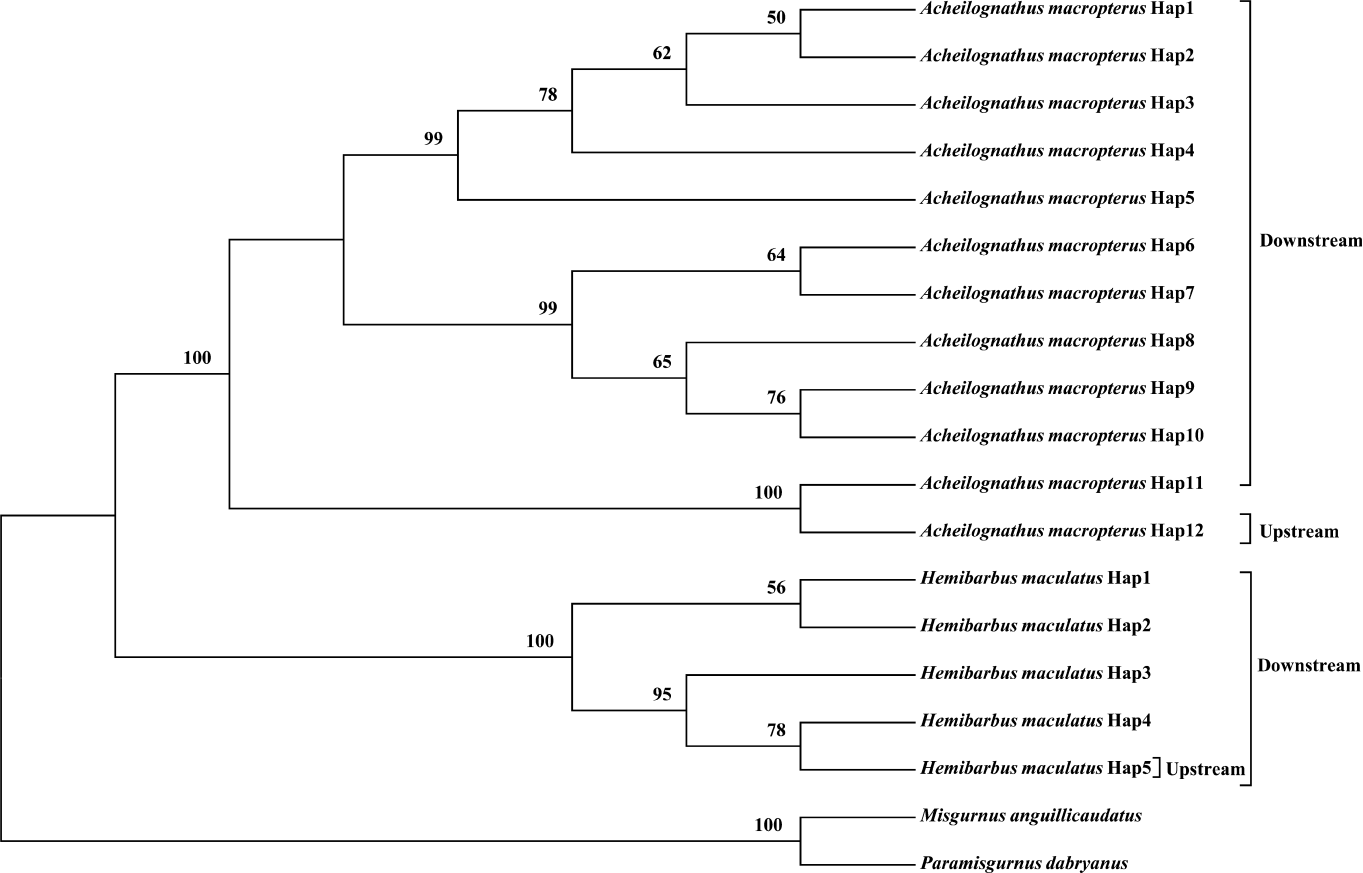
Neighbor‐joining tree based on the K2P of the full haplotypes for *Acheilognathus macropterus* and *Hemibarbus maculatus*. Bootstrap values greater than 50 are shown.

In this study, eight subfamilies of cyprinidae (Xenocyprinae, Cultrinae, Danioninae, Gobioninae, Cyprininae, Leuciscinae, Hypophthalmichthyinae, and Acheilognathinae), were characterized by DNA barcoding. The NJ tree based on the p‐distance model was not shown because of the same topology and similar bootstrap values to those of the K2P model. With the exception of *Sarcocheilichthys sinesis*, the remaining species shared similar topologies in the NJ and ML trees, but all species formed distinct clusters in the trees (Fig. 3). For species with two or more haplotypes, all the haplotypes were associated with their conspecifics in the monophyletic clades with high support (98% bootstrap in NJ, 91% in ML and 98% probability in MrBayes). However, not all the species from the same subfamily clustered together in the three trees. In total, 11 Cultrinae species, three Cyprininae species, two Acheilognathinae species, two Hypophthalmichthyinae species and one Danioninae species clustered together in the three trees. Moreover, two Xenocyprinae species and three Leuciscinae species clustered together in the Bayesian tree but not in the NJ and ML trees (Fig. 4). The remaining species from the same subfamilies formed different clusters with other subfamilies (Figs. 3, 4). Full haplotype trees for *A. macropterus* and *H. Maculatus* are shown in Figure 5.

### Species genetic diversity analyses

Six of the 10 native species showed few haplotypes. Only one haplotype was observed for *Coreius heterodon* (*n* = 23) and *Rhinogobio typus* (*n* = 16), two for *Pseudobrama simoni* (*n* = 26), three for *S. gracilicaudatus* (*n* = 9) and *Ancherythroculter nigrocauda* (*n* = 9), and four for *H. tchangi* (*n* = 18). The *h* and *π* values were less than 0.556 and 0.0018, respectively. The values of the remaining four species had not been calculated, owing to the small sample sizes (less than five samples; *P. rabaudi* [*n* = 1], *A. kurematsui* [*n* = 2], *Rhinogobio cylindricus* [*n* = 5], and *Abbottina obtusirostris* [*n* = 2]) (Table 3).

**Table 3:** DNA polymorphism of 35species

Species	Whole dataset			Upstream of TG			Downstream of TG			Shared haplotypes
	*H*/*N*/*n*	*h* (SE)	*π* (SE)	*H*/*N*/*n*	*h* (SE)	*π* (SE)	*H*/*N*/*n*	*h* (SE)	*π* (SE)	
*Abbottina obtusirostris*	2/2/2									
*Procypris rabaudi*	1/1/1									
*Rhinogobio cylindricus*	2/5/1									
*Ancherythroculter kurematsui*	1/2/1									
*Coreius heterodon*	1/23/4	0	0							
*Rhinogobio typus*	1/16/3	0	0							
*Saurogobio gracilicaudatus*	3/9/1	0.417 (0.064)	0.0018 (0.0003)							
*Ancherythroculter nigrocauda*	3/9/1	0.556 (0.055)	0.0010 (0.0001)							
*Hemiculter tchangi*	4/18/2	0.529 (0.028)	0.0013 (0.0001)							
*Pseudobrama simoni*	2/26/3	0.077 (0.014)	0.0001 (0.0000)	2/16/2	0.125 (0.027)	0.0002 (0.0001)	1/10/1	0	0	1
*Abbottina rivularis*	3/25/6	0.293 (0.022)	0.0005 (0.0000)	3/17/4	0.412 (0.033)	0.0007 (0.0000)	1/8/2	0	0	1
*Carassius auratus*	11/31/5	0.856 (0.007)	0.0038 (0.0000)	3/7/2	0.524 (0.079)	0.0014 (0.0003)	9/24/3	0.866 (0.008)	0.0041 (0.0000)	1
*Culter alburnus*	4/13/3	0.744 (0.025)	0.0021 (0.0000)	4/11/2	0.745 (0.030)	0.0023 (0.0000)	2//2/1	1.000 (0.354)	0.0016 (0.0006)	2
*Cyprinus carpio*	14/38/6	0.871 (0.006)	0.0034 (0.0000)	3/4/2	0.833 (0.111)	0.0032 (0.0006)	12/34/4	0.875 (0.007)	0.0034 (0.0000)	1
*Saurogobio dabryi*	22/52/7	0.933 (0.002)	0.0082 (0.0000)	13/27/3	0.909 (0.007)	0.0090 (0.0000)	15/25/4	0.897 (0.009)	0.0059 (0.0002)	6
*Squalidus argentatus*	6/10/3	0.889 (0.024)	0.0045 (0.0003)	5/8/2	0.893 (0.030)	0.0041 (0.0003)	2/2/1	1.000 (0.354)	0.0064 (0.0023)	1
*Hemiculter bleekeri*	24/56/4	0.847 (0.005)	0.0026 (0.0000)	12/32/2	0.817 (0.009)	0.0026 (0.0000)	16/24/2	0.895 (0.012)	0.0025 (0.0000)	4
*Acheilognathus macropterus*	12/19/4	0.947 (0.007)	0.0202 (0.0015)	1/2/1	0	0	11/17/3	0.941 (0.009)	0.0174 (0.0006)	0
*Hemibarbus maculatus*	5/20/7	0.511 (0.029)	0.0087 (0.0007)	1/12/4	0	0	5/9/2	0.861 (0.029)	0.0169 (0.0013)	1
*Culter mongolicus*	2/7/2	0.286 (0.074)	0.0005 (0.0001)							
*Cultrichthys erythropterus*	7/13/3	0.795 (0.030)	0.0023 (0.0001)							
*Hemiculter leucisculus*	13/31/4	0.772 (0.013)	0.0027 (0.0000)							
*Pseudolaubuca sinensis*	3/48/2	0.566 (0.005)	0.0010 (0.0000)							
*Hypophthalmichthys molitrix*	3/14/2	0.385 (0.040)	0.0015 (0.0002)							
*Sarcocheilichthys sinensis*	3/7/1	0.714 (0.048)	0.0050 (0.0003)							
*Squaliobarbus curriculus*	2/20/2	0.100 (0.020)	0.0002 (0.0000)							
*Xenocypris argentea*	7/19/2	0.608 (0.029)	0.0017 (0.0001)							
*Parabramis pekinensis*	3/7/2	0.667 (0.060)	0.0014 (0.0002)							
*Elopichthys bambusa*	2/4/1									
*Ctenopharyngodon idellus*	1/5/1									
*Hypophthalmichthys nobilis*	1/3/2									
*Opsariichthys bidens*	1/1/1									
*Pseudolaubuca engraulis*	3/5/2									
*Rhodeus ocellatus*	1/1/1									
*Saurogobio dumerili*	1/1/1									

Native species with underline. Shared haplotypes: haplotypes were shared between the upstream and downstream populations; *H*, number of haplotypes; *N*, number of specimens; *n*, number of sampling sites; *h*, haplotype diversity; *π*, nucleotide diversity; SE, standard error; TG, Three Gorges.

For the remaining non‐native species, 18 species with sample sizes of more than five samples had *h* values that ranged from 0.100 (*Squaliobarbus curriculus*) to 0.947 (*A. macropterus*) and *π* values that ranged from 0.0002 (*S. curriculus*) to 0.0202 (*A. macropterus*) (Table 3).

Ten species with overall nucleotide diversities greater than 0.00% were selected to evaluate the differences in the genetic diversity of both the upstream and downstream populations (Table 3). For *A. macropterus*, no shared haplotypes were detected between the upstream and downstream populations. However, shared haplotypes were detected for the other nine species: only one each for *Abbottina rivularis*,* Carassius auratus*,* Cyprinus carpio*,* P. simoni*,* Squalidus argentatus*, and *H. maculatus*; two for *Culter alburnus*; four for *Hemiculter bleekeri*; and six for *Saurogobio dabryi* (Table 3).

## Discussion

### Barcoding success

DNA barcoding using the COI gene as a tag for identifying species, especially fish species, has recently attracted attention (McCusker et al. 2013; Knebelsberger et al. 2014). One of the primary reasons for the selection of COI as the gold standard barcode gene is the typical pattern of variation observed for numerous species, with both marked divergence and a lack of overlap between the intraspecific (i.e., between individuals of the same species) and interspecific (i.e., between individuals of different species) genetic distances (Hebert et al. 2003a,b). The intraspecific genetic distances based on K2P are usually low (below 1%) and are rarely greater than 2% across a broad range of taxa (Hebert et al. 2003a,b), including fish (Ward 2012). Of the 35 species investigated in this study, only two had interspecific differences based on K2P and p‐distance that exceeded 2% (*A. macropterus* and *H. maculatus*). Species sampled from several regions showed less than 2% sequence diversity, which indicated no increase in genetic variability relative to species from a single region. The delimitation of species based on the comparison of mean intraspecific and interspecific genetic distances is a primary concern for barcoding studies. A 10‐fold sequence difference between the average interspecific and the average intraspecific differences has been suggested as the standard COI threshold for animal species identification (Hebert et al. 2004). In our present study, the values were 19.67 for the K2P distance and 18.33 for the p‐distance.

The use of the mean instead of the smallest interspecific distance exaggerates the size of the “barcoding gap” and leads to misidentification. One approach to detect the barcoding gap is to determine the overlap between the lowest interspecific and the highest intraspecific genetic distances (Meier et al. 2008). In this study, we found no such overlap in any of the 35 species, and the barcoding gaps ranged from 1.49 (*Hemiculter leucisculus*) to 18.09 (*R. ocellatus*) for K2P and from 1.44 (*H. leucisculus*) to 15.87 (*R. ocellatus*) for the p‐distance.

In our sequence library comprising 35 fish species from the midstream of the Yangtze River, all sequence haplotypes of the same species formed high bootstrap‐supported clusters in the three trees. Thus, the DNA barcoding was 100% successful, which was higher than the 93% success rate reported for Canadian freshwater fish (Hubert et al. 2008) and the 90% success rate reported for North American freshwater fish (April et al. 2011). The high discrimination power of DNA barcoding in our data set may have occurred because most of the genera were represented by only one species; therefore, the number of closely related congeners was quite low. In conclusion, this library is a highly valuable and reliable identification tool for fisheries research on economically important species at all developmental stages, which will guarantee a sustainable exploitation of cyprinid natural resources in the Yangtze River.

### Cryptic species

In this study, two of the 35 species (*A. macropterus* and *H. maculatus*) had high intraspecific K2P distances of both 3.81% and intraspecific p‐distances of 3.54% and 3.70%, respectively; all other species were below 2%. Interestingly, both of these species showed high haplotype diversity downstream of the TG but only a single haplotype upstream. Judging from the overall haplotype diversity values and for these two species, in fish isolated from the downstream portion of the Yangzte River, the upstream haplotype was rare in *A. macropterus* but common in *H. maculatus*. Moreover, the full haplotype tree for these two species showed that the upstream haplotype in each species fell outside the main haplotype cluster. Thus, these two species might be cryptic species, thus potentially explaining the high intraspecific diversity.

### Genetic diversity

The genetic diversity of fish may reflect human disturbances and environmental disruptions that are likely driving the population decline. Additionally, genetic factors can speed up the extinction process once a population becomes very small (Westemeier et al. 1998). In this study, the COI barcode diversity was found to be low (≤0.50%), especially for the 10 native species (≤0.18%); the exceptions were *H. maculatus* (0.87%), *A. macropterus* (2.02%), and *S. dabryi* (0.82%). For the 10 species divided into two populations (upstream of TG and downstream of TG), no COI haplotypes for one species, and only one for six species, were shared by the upstream and downstream populations (Table 3). Therefore, the data are suggestive of population structure which may have occurred in the upstream versus the downstream populations. However, these results are only suggestive and a more dedicated study of population structure using appropriate markers should be conducted in the future. If such structure is eventually confirmed through more detailed study, then it is vital that policy actions for conservation should be taken immediately, especially for native species.

## Conflict of interest

None declared.

## Supporting information


**Table S1.** Information of samples used in this study. Species with bold font represented only one sample.
