# Supplementary material for: DNA barcoding and evaluation of genetic diversity in Cyprinidae fish in the midstream of the Yangtze River
Source: Ecol Evol. 2016 Mar 17;6(9):2702–13. doi: 10.1002/ece3.2060 (PMC4798831; doi:10.1002/ece3.2060)
Supplement: Supplementary file 1 — Table S1. Information of samples used in this study. Species with bold font represented only one sample. [file ECE3-6-2702-s001.docx]

**Table S1** Information of samples used in this study. Species with bold font represented only one sample.

| Species | Province | Locality | No. of specimens | GenBank accession No |
| --- | --- | --- | --- | --- |
| *Abbottina obtusirostris* | Sichuan | Suji, Leshan | 1 | KR861721- KR861722 |
|  | Sichuan | Wutongqiao, Leshan | 1 |  |
| *Abbottina rivularis* | Sichuan | Suji, Leshan | 6 | KR861723- KR861747 |
|  | Sichuan | Wutongqiao, Leshan | 9 |  |
|  | Sichuan | Rongzhou, Yibin | 1 |  |
|  | Jiangxi | Hukou, Jiujiang | 5 |  |
|  | Jiangxi | Duchang, Jiujiang | 3 |  |
|  | Chongqing | Mudong | 1 |  |
| *Carassius auratus* | Chongqing | Mudong | 3 | KR861778- KR861808 |
|  | Sichuan | Hejiang, Luzhou | 4 |  |
|  | Jiangxi | Hukou, Jiujiang | 6 |  |
|  | Jiangxi | Duchang, Jiujiang | 3 |  |
|  | Jiangxi | Xingzi, Jiujiang | 15 |  |
| *Coreius heterodon* | Hubei | Jiangxia, Wuhan | 3 | KR861809- KR861831 |
|  | Hunan | Dongting lake, Yueyang | 11 |  |
|  | Chongqing | Mudong | 1 |  |
|  | Jiangxi | Hukou, Jiujiang | 8 |  |
| *Ctenopharyngodon idellus* | Jiangxi | Hukou, Jiujiang | 5 | KR861832- KR861836 |
| *Culter alburnus* | Chongqing | Mudong | 9 | KR861837- KR861849 |
|  | Sichuan | Hejiang, Luzhou | 2 |  |
|  | Jiangxi | Duchang, Jiujiang | 2 |  |
| *Culter mongolicus* | Sichuan | Hejiang, Luzhou | 5 | KR861850- KR861856 |
|  | Chongqing | Mudong | 2 |  |
| *Cultrichthys erythropterus* | Jiangxi | Duchang, Jiujiang | 7 | KR861857- KR861869 |
|  | Jiangxi | Xingzi, Jiujiang | 4 |  |
|  | Jiangxi | Hukou, Jiujiang | 2 |  |
| *Cyprinus carpio* | Sichuan | Hejiang, Luzhou | 1 | KR861870- KR861907 |
|  | Jiangxi | Hukou, Jiujiang | 16 |  |
|  | Jiangxi | Duchang, Jiujiang | 6 |  |
|  | Jiangxi | Xingzi, Jiujiang | 11 |  |
|  | Chongqing | Mudong | 3 |  |
|  | Hubei | Jiangxia, Wuhan | 1 |  |
| *Elopichthys bambusa* | Hunan | Dongting lake, Yueyang | 4 | KR861908- KR861911 |
| *Hemiculter bleekeri* | Sichuan | Hejiang, Luzhou | 14 | KR861932- KR861987 |
|  | Chongqing | Mudong | 18 |  |
|  | Jiangxi | Hukou, Jiujiang | 12 |  |
|  | Jiangxi | Xingzi, Jiujiang | 12 |  |
| *Hemiculter leucisculus* | Sichuan | Wutongqiao, Leshan | 1 | KR861988- KR862018 |
|  | Jiangxi | Duchang, Jiujiang | 14 |  |
|  | Jiangxi | Xingzi, Jiujiang | 10 |  |
|  | Jiangxi | Hukou, Jiujiang | 6 |  |
| *Hypophthalmichthys molitrix* | Jiangxi | Hukou, Jiujiang | 13 | KR862037- KR862050 |
|  | Hubei | Jiangxia, Wuhan | 1 |  |
| *Hypophthalmichthys nobilis* | Chongqing | Mudong | 1 | KR862051- KR862053 |
|  | Jiangxi | Hukou, Jiujiang | 2 |  |
| ***Opsariichthys bidens*** | **Hunan** | **Dongting lake, Yueyang** | **1** | **KR862054** |
| ***Procypris rabaudi*** | **Sichuan** | **Hejiang, Luzhou** | **1** | **KR862062** |
| *Pseudobrama simoni* | Sichuan | Hejiang, Luzhou | 15 | KR862063- KR862088 |
|  | Chongqing | Mudong | 1 |  |
|  | Jiangxi | Duchang, Jiujiang | 10 |  |
| *Pseudolaubuca engraulis* | Jiangxi | Hukou, Jiujiang | 1 | KR862089- KR862093 |
|  | Jiangxi | Duchang, Jiujiang | 4 |  |
| *Pseudolaubuca sinensis* | Sichuan | Hejiang, Luzhou | 34 | KR862094- KR862141 |
|  | Chongqing | Mudong | 14 |  |
| *Rhinogobio typus* | Hunan | Dongting lake, Yueyang | 6 | KR862142- KR862157 |
|  | Chongqing | Mudong | 1 |  |
|  | Hubei | Jiangxia, Wuhan | 9 |  |
| ***Rhodeus ocellatus*** | **Chongqing** | **Mudong** | **1** | **KR862163** |
| *Sarcocheilichthys sinensis* | Hunan | Dongting lake, Yueyang | 7 | KR862164- KR862170 |
| *Saurogobio dabryi* | Hunan | Dongting lake, Yueyang | 11 | KR862171- KR862222 |
|  | Sichuan | Hejiang, Luzhou | 12 |  |
|  | Sichuan | Rongzhou, Yibin | 6 |  |
|  | Chongqing | Mudong | 9 |  |
|  | Jiangxi | Duchang, Jiujiang | 6 |  |
|  | Jiangxi | Hukou, Jiujiang | 4 |  |
|  | Jiangxi | Xingzi, Jiujiang | 4 |  |
| ***Saurogobio dumerili*** | **Jiangxi** | **Hukou, Jiujiang** | **1** | **KR862223** |
| *Squalidus argentatus* | Chongqing | Mudong | 4 | KR862233- KR862242 |
|  | Jiangxi | Hukou, Jiujiang | 2 |  |
|  | Sichuan | Hejiang, Luzhou | 4 |  |
| *Squaliobarbus curriculus* | Hunan | Dongting lake, Yueyang | 19 | KR862243- KR862262 |
|  | Jiangxi | Duchang, Jiujiang | 1 |  |
| *Saurogobio gracilicaudatus* | Hunan | Dongting lake, Yueyang | 9 | KR862224- KR862232 |
| *Acheilognathus macropterus* | Chongqing | Mudong | 2 | KR861749- KR861765 |
|  | Jiangxi | Hukou, Jiujiang | 2 |  |
|  | Jiangxi | Duchang, Jiujiang | 3 |  |
|  | Jiangxi | Xingzi, Jiujiang | 12 |  |
| *Hemibarbus maculatus* | Hunan | Dongting lake, Yueyang | 8 | KR861912- KR861931 |
|  | Sichuan | Suji, Leshan | 5 |  |
|  | Sichuan | Wutongqiao, Leshan | 4 |  |
|  | Sichuan | Hejiang, Luzhou | 1 |  |
|  | Chongqing | Mudong | 1 |  |
|  | Jiangxi | Xingzi, Jiujiang | 1 |  |
| *Xenocypris argentea* | Jiangxi | Duchang, Jiujiang | 7 | KR862263- KR862281 |
|  | Jiangxi | Hukou, Jiujiang | 12 |  |
| *Hemiculter tchangi* | Chongqing | Mudong | 12 | KR862019- KR862036 |
|  | Sichuan | Hejiang, Luzhou | 6 |  |
| *Parabramis pekinensis* | Hunan | Dongting lake, Yueyang | 1 | KR862055- KR862061 |
|  | Jiangxi | Duchang, Jiujiang | 6 |  |
| *Ancherythroculter nigrocauda* | Chongqing | Mudong | 9 | KR861769- KR861777 |
| *Rhinogobio cylindricus* | Sichuan | Hejiang, Luzhou | 5 | KR862158- KR862162 |
| *Ancherythroculter kurematsui* | Sichuan | Hejiang, Luzhou | 2 | KR861767- KR861768 |
| **Total** |  |  | **561** |  |
